# PEGylated Liposomal Methyl Prednisolone Succinate does not Induce Infusion Reactions in Patients: A Correlation Between in Vitro Immunological and in Vivo Clinical Studies

**DOI:** 10.3390/molecules25030558

**Published:** 2020-01-28

**Authors:** Yaelle Bavli, Bing-Mae Chen, Steve R. Roffler, Marina A. Dobrovolskaia, Eldad Elnekave, Shifra Ash, Yechezkel Barenholz, Keren Turjeman

**Affiliations:** 1 Laboratory of Membrane and Liposome Research, Department of Biochemistry, Institute for Medical Research Israel-Canada, Hebrew University-Hadassah Medical School, Jerusalem 9112102, Israel; yaellef@ekmd.huji.ac.il (Y.B.); kerenbarhum@gmail.com (K.T.); 2 Institute of Biomedical Sciences, Academia Sinica, Taipei 11529, Taiwan; bingmae@ibms.sinica.edu.tw (B.-M.C.); sroff@ibms.sinica.edu.tw (S.R.R.); 3 Nanotechnology Characterization Laboratory, Cancer Research Technology Program, Frederick National Laboratory for Cancer Research sponsored by the National Cancer Institute, Frederick, MD 21702, USA; marina@mail.nih.gov; 4 Davidoff Cancer Institute, Rabin Medical Center, Petach Tikva 4941492, Israel; 5 Rina Zaizov Pediatric Hematology Oncology Division, Schneider Children’s Medical Center of Israel, Petach Tiqva, Tel Aviv University, Tel Aviv, Israel 4920235, Israel; Shifraa@clalit.org.il

**Keywords:** PEGylated nanodrugs, hypersensitive reactions, anti-PEG antibodies, complement activation, liposomal steroids, IgG4 related disease

## Abstract

PEGylated nanomedicines are known to induce infusion reactions (IRs) that in some cases can be life-threatening. Herein, we report a case study in which a patient with rare mediastinal and intracardiac IgG4-related sclerosing disease received 8 treatments of intravenously administered PEGylated liposomal methylprednisolone-succinate (NSSL-MPS). Due to the ethical requirements to reduce IRs, the patient received a cocktail of premedication including low dose of steroids, acetaminophen and H2 blockers before each infusion. The treatment was well-tolerated in that IRs, complement activation, anti-PEG antibodies and accelerated blood clearance of the PEGylated drug were not detected. Prior to the clinical study, an in vitro panel of assays utilizing blood of healthy donors was used to determine the potential of a PEGylated drug to activate complement system, elicit pro-inflammatory cytokines, damage erythrocytes and affect various components of the blood coagulation system. The overall findings of the in vitro panel were negative and correlated with the results observed in the clinical phase.

## 1. Introduction 

The advantages of nanodrugs derive from their physicochemical properties, especially their nano-size, their ability to passively target tumors [1], inflamed [2,3] and bacteria-infected [4] tissues as well as their ability to carry both hydrophobic and hydrophilic drugs. However, there are some major barriers and obstacles to overcome to meet the high expectation from nanomedicines [5]. One of such obstacles is the increase in the number of reports of interactions between nanoparticles and the immune system, which shed light on the risks associated with their use [6,7,8]. Of special relevance is the relatively high occurrence of “infusion reactions” (IRs)-also referred to as hypersensitivity reactions (HSR)-highlighting the importance of early and reliable estimation of the potential of such immune responses to occur. The symptoms of IRs include, but are not limited to, skin flushing, chest tightness, hypotension, dyspnea, facial swelling, headaches, chills and back pain. In most cases the reactions are mild, transient and readily controllable; in rare cases, they can manifest with rapid and severe cardiopulmonary compromise [9]. The drug delivery systems and drug products capable of inducing IRs are very diverse and include liposomes, micelles, antibodies and enzymes. While the majority of reactions have been observed in non-PEGylated molecules, PEGylated proteins as well as PEGylated micellar and liposomal formulations have also been observed to trigger acute IRs [10]. Unlike antigens causing type I immediate hypersensitivity reactions, the IRs to PEGylated drug products are not mediated by drug-specific IgEs; instead, cytokine and complement (C) activation are involved in these responses [9]. 

In the case of PEGylated nanodrugs, there are further indications that these adverse effects may also be triggered by the presence of anti-PEG antibodies (IgM and/or IgG) in the patient’s blood, either pre-existing [11,12,13,14] or elicited by the administration of the PEGylated agent [14,15,16]. In a recent study aiming at improving our understanding of the role of anti-PEG antibodies in complement activation by Doxil in vitro [17], we showed the complexity of this activity. Under in vitro conditions, some anti-PEG antibodies contribute to complement activation by Doxil^®^. However, such contribution needs to be considered in the context of other factors, including, but not limited to, antibody class, type, clonality, epitope specificity, affinity, and titer. In a recent perspective in Nature Nanotechnology [9], we suggest that the relevance of animal models and in vitro characterization of the PEGylated nano-drug remain unclear and require more human data [9].

An earlier study of the Han Chinese population residing in Taiwan assessed the prevalence of pre-existing anti-PEG antibodies in this population, and revealed that more than 44% of the population had in their plasma either circulating anti-PEG IgG or IgM (25.7% and 27.1% respectively), and that 8.4% of the population were positive for both types of antibodies without any prior treatment with a PEGylated drug [12]. Such high prevalence cannot be ignored with respect to potential IRs. There is an indication from non-human mammalian species [18,19,20,21] that such antibodies may also lead to the Accelerated Blood Clearance (ABC) phenomenon, which results in fast clearance of PEGylated liposomes at subsequent drug administration [22]. This was also observed more recently in patients who received PEGylated nanodrugs [14,15,16]. Together, these adverse immunological responses may cancel or reduce the therapeutic benefits of the administered nanodrugs. Unfortunately, the in vitro tests that are available now can give indication of the immunogenic potential of the drug, and therefore on the risk of IRs, but not on the incidence or their magnitude once the drug is administered to patients. Therefore, it is of crucial importance to be able to find a reliable way to predict the risks of IRs as early as possible. In order to evaluate the in vitro-in vivo correlation that may enable prediction of IRs in patients, we correlate the in vitro interaction of a nano-drug in normal serum with all the aspects of reactogenicity of this nano-drug in a patient. 

As the nano-drug we used a 80 nm formulation of PEGylated sterically stabilized nano-liposomes (NSSL) remotely loaded with the amphipathic weak acid, steroid prodrug methylprednisolone hemisuccinate (MPS), to produce the steroidal nano-drug NSSL-MPS. The MPS was remotely loaded using a trans-membrane calcium acetate gradient [23]. The NSSL-MPS has been shown to have high therapeutic efficacy in several animal models of inflammatory diseases including multiple sclerosis, rheumatoid arthritis, lupus, Duchenne muscular dystrophy, local inflammation and cerebral malaria [23,24,25,26,27,28]. These animal studies proved that the small size of the liposomes (~80 nm) combined with the PEG corona promote size-dependent passive targeting and drug accumulation in the diseased tissue. The accumulation of drug-loaded nano-liposomes at the inflamed tissue, followed by slow, zero-order drug release there explain their superior therapeutic efficacy as well as improvement in the inflamed tissue pathology and reduced toxicity following long-term treatment. 

Herein, we report a clinical case, in which NSSL-MPS were administered to a 21-year old patient with mediastinal and intra-cardiac IgG4-related sclerosing disease (IgG4 RSD) under a compassionate-use (“first in human”) protocol. IgG4 RSD is a rare disease that can manifest in myriad organ systems [29] by infiltration of “masses” composed mostly of lymphocytes and fibroblasts that can result in multi-organ dysfunction and even mortality [30,31]. Elevated serum IgG4 concentrations are observed in the majority of patients [32]. IgG4 RSD is a rare disease and its exact prevalence is hard to determine but in Japan, where IgG4 RSD has been most extensively studied, a prevalence of 0.28–1.08 per 100,000 individuals has been estimated for all forms of the disease [33]. The patient described herein was diagnosed at age 18 with IgG4 RSD, with over >50% of plasma cells staining positively for IgG4 and elevated serum levels of IgG4 (480 vs. normal 3.9–86 mg/dL). Prior to this administration a comprehensive in vitro immunoassay panel was conducted to estimate the potential risk of infusion reactions to this PEGylated liposomal product. The overall findings of this in vitro panel was negative, suggesting a low risk of the IRs in response to the NSSL-MPS.

Glucocorticoids are considered the therapy of choice for treating IgG4 RSD and in this case, previous successive treatments including oral steroids proved only partially effective. High dose pulsed IV Solumedrol and selective intra-arterial Solumedrol were correlated with a drop of serum IgG4 concentrations to within normal levels but failed to achieve a decrease in mass size; and follow up MRI’s demonstrated progressive infiltration of the mass into the heart and the superior gastric wall. The results supported further use of Solumedrol as a therapeutic agent, although dose-dependent toxicities prevented the continuation of systemic use, while repetitive intra-arterial treatments were limited by the risks inherent in invasive angiography. 

At this point, a systemic solution was sought which would maximize therapeutic efficacy of Solumedrol while minimizing systemic exposure. Preliminary testing indicated that the target mass exhibited hyper-vascularity, which, together with the inflammatory tissue constitution, made it an excellent candidate for nano-liposomal targeting and accumulation. Therefore, the treatment of the patient by NSSL-MPS in the scope of a compassionate use was approved by the Schneider Hospital Local Helsinki committee and Israel Ministry of Health (number: 02-61-16-RMC) and informed written consent was obtained from the patient before the beginning of treatment. 

This study was aimed to put into practice our knowledge of the different arms that can trigger IRs in patients following the administration of PEGylated liposomes well characterized for their in vitro reactogenicity and test before, during and after each treatment the patient’s plasma and serum for signs of immune reaction and correlate with the apparition of clinical signs if any would manifest. 

## 2. Results

### 2.1. In Vitro Immune-Compatibility of NSSL-MPS

**Figure 1:**
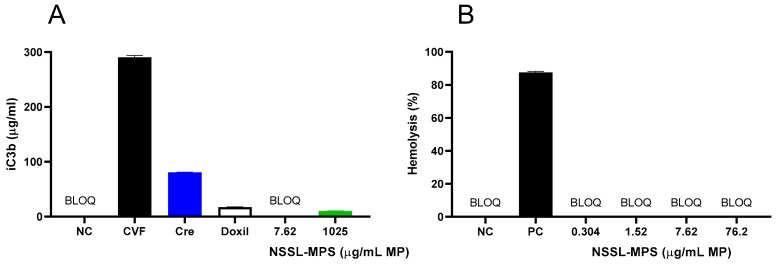
Evaluation of in vitro activation of the complement system and hemolytic properties of PEGylated liposomal methylprednisolone-succinate NSSL-MPS. (**A**) Different concentrations of NSSL-MPS were incubated in human plasma for 30 min and the increase in iC3b was measured and compared to iC3b activation by Cobra Venom Factor (CVF) (**B**)**.** Different concentrations of NSSL-MPS were incubated in whole blood and the free hemoglobin released by damaged erythrocytes was quantified. BLOQ, below limit of quantitation; Cre, Cremophor-EL; CVF, Cobra Venom Factor; NC, Negative Control; PC, Positive Control (Triton-X-100). BLOQ is < 0.13 µg/mL.

**Figure 2:**
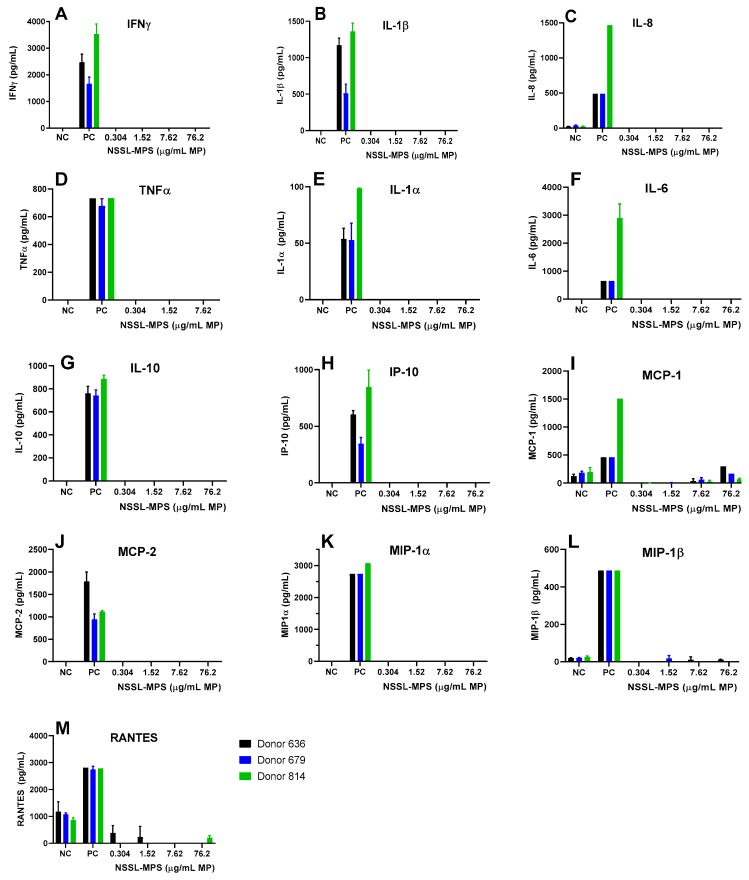
Individual cytokines/chemokines/interferon release tests in the presence of the NSSL-MPS formulation. NSSL-MPS at different concentrations (0.304, 1.52, 7.62 or 76.2 µg/mL MP) were incubated for 24 h in whole human blood from at least 3 different healthy donors. After incubation, the supernatant was tested for each relevant signaling molecule with a corresponding ELISA assay. NC, Negative Control; PC, Positive Control, 20 ng/mL of *E.coli K12* LPS.

**Figure 3:**
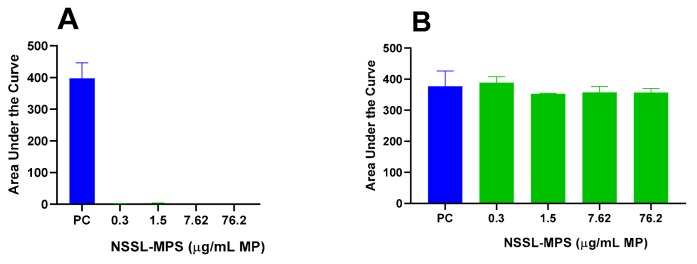
NSSL-MPS effect on platelet aggregation and collagen-induced platelet aggregation. The capacity of NSSL-MPS to induce platelet aggregation (**A**) and modify collagen-induced platelet aggregation (**B**) were assessed. Briefly, NSSL-MPS at different concentrations (0.304, 1.52, 7.62 or 76.2 µg/mL MPS) were incubated in platelet-rich plasma for 15 min at 37 °C. Platelet aggregation was monitored in real time using light transmission aggregometry. AUC, Area Under the Curve; PC, Positive Control (positive control is collagen).

**Figure 4:**
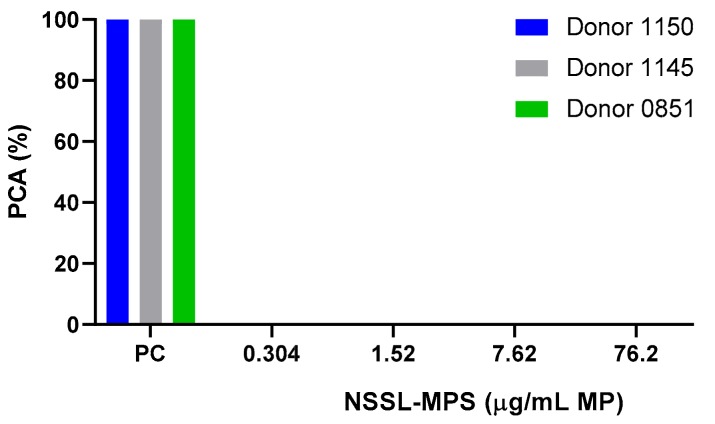
Leucocyte pro-coagulant activity in the presence of NSSL-MPS. Different concentrations of NSSL-MPS were tested for their ability to induce leukocyte pro-coagulant activity. Briefly, peripheral blood mononuclear cells (PBMC) isolated from healthy blood donor volunteers and HL-60 cells were incubated (24 h for PBMC and 5 h for HL-60 cells) with NSSL-MPS at 0.304, 1.52, 7.62 or 76.2 µg/mL MPS. Isolated cells from the different conditions were then used to initiate plasma coagulation. PC, Positive Control; PCA, Pro-Coagulant Activity.

First, to assess the likelihood of the NSSL-MPS formulation to induce infusion reactions in vivo, we performed a panel of in vitro tests prior to NSSL-MPS administration to the patient. In vitro complement activation was assessed by incubating NSSL-MPS in plasma of healthy human donors followed by the measurement of an increase in the split product iC3b. Two materials known to activate the complement system in patients (Doxil® and Cremophor-EL®) were used as additional positive controls. As shown in Figure 1A, in vitro incubation of NSSL-MPS in human plasma at the clinically relevant concentration did not result in detectable complement activation. At the very high concentration, low levels of iC3b were detected but were lower than that induced by Doxil®. NSSL-MPS was not hemolytic at all tested concentrations (Figure 1B). Inhibition enhancement controls composed of the positive control supernatant spiked with the nanoparticle sample did not reveal an interference of the nanoparticle with in vitro assays. Another in vitro test assessed the release of 13 different cytokines in whole blood cultured in the presence of NSSL-MPS. This test is considered a reliable test for the prediction of cytokines storms, a severe condition characterized by the overproduction of inflammatory cytokines, chemokines and interferons in response to certain pathogens or therapeutics. This test assessed the presence of the following cytokines: IFNγ, TNFα, IL-1α, IL-1β, IL-6, IL-8, IL-10, IP-10 (Interferon gamma-induced protein 10), MCP-1 (Monocyte chemoattractant protein), MCP-2, MIP-1α (Macrophage inflammatory protein), MIP-1β and RANTES (Regulated on Activation, Normal T Cell Expressed and Secreted). The results are presented in Figure 2 and show that NSSL-MPS did not induce the production of any of the tested cytokines in blood cultures derived from several healthy donors. In addition, the NSSL-MPS did not induce platelet aggregation, did not modify collagen-induced platelet aggregation (Figure 3), did not induce leukocyte pro-coagulant activity (Figure 4), and did not affect plasma coagulation time (Figure S1 presented in Supplementary Materials). The in vitro panel, therefore, suggested that the risk of NSSL-MPS to induce IRs is minimal and does not exceed that to Doxil, which is successfully used in the clinic.

### 2.2. Clinical Study

It is well established that clinical signs of infusion reactions vary between patients and can include one or more of the following symptoms: flushing, urticaria, rash, pruritus, shortness of breath, asthma, bronchospasm, apnea, hypotension, tachycardia, facial swelling, tightness in the chest and throat, headaches, chills, chest pain, back pain, fever, cyanosis or syncope. The more rapidly a reaction develops, the more severe it is likely to be [34,35].

We monitored vital signs of the patient during and after each NSSL-MPS infusion to be able to detect early signs of infusion reaction. Throughout the treatments (during the two courses of treatment, a total of 8 infusions), the patient showed no clinical signs of infusion reactions. The only reported adverse effects were mild headaches after the 3rd and 4th administration during the first course and the 2nd administration of the second course (7th administration).

A very common side-effect of steroid treatment (especially prednisone) is corticosteroid induced hyperglycemia [36]. Therefore we measured blood glucose levels (non-fasting) during and after each treatment, and the values remained within normal limits (Figure S2 in Supplementary Materials) despite 2 measurements that were performed 1–2 h post prandial (on day 8, 24 h after the second treatment and on day 30, 24 h after treatment 5). Another common side-effect of steroid treatment are changes in blood count. Follow up (details in Figure S3) shows transient spikes of leukocytes (white blood cells, WBC) of up to 20.2 × 10^3^/µL WBC 24–48 h after the treatments that returned to baseline within 2–6 days (A). Figure S3 (B) depicts the mild and transient decrease observed in lymphocytes, and Figure S3 (C) the increase in neutrophils mirroring the decrease in lymphocytes occurring after NSSL-MPS infusions. All blood counts usually returned to baseline within 6–7 days.

### 2.3. In Vivo Complement Activation and Inflammatory Disease Markers

**Figure 5:**
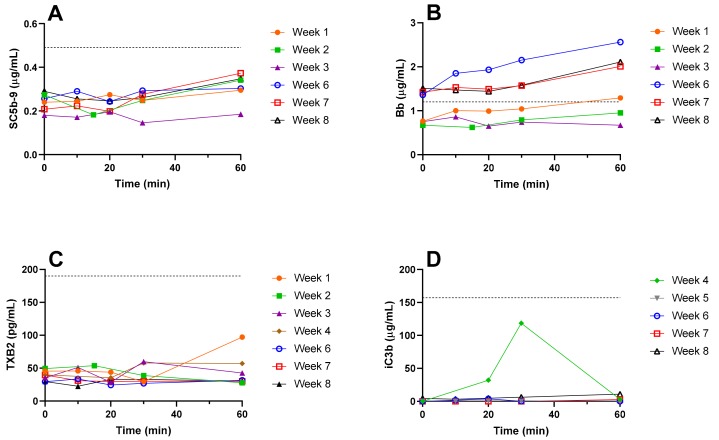
Markers of in vivo complement activation in plasma. Immediately before the beginning of NSSL-MPS infusion (0) and 10, 20, 30 and 60 min after the beginning of the infusion, a 2 mL sample of blood was withdrawn in K_2_EDTA blood collection tubes. The blood was kept on ice and centrifuged at 960 x*g* for 10 min at 4 °C as soon as possible after collection. Immediately after centrifugation, the plasma was collected and frozen on dry ice. The following markers of complement activation were assayed using commercial kits (detailed in the Materials section): SC5b-9 (**A**), Bb (**B**), TXB2 (**C**) and iC3b (**D**). The dash line indicates the upper limit of normal values. TXB, Thromboxane.

Plasma samples were collected from the patient during the NSSL-MPS infusion (over a period of an hour) and great care was taken to keep them cold at all times to avoid artifact activation of complement *ex vivo.* Initially (treatments 1–3), the markers of complement activation tested in the plasma of the patient immediately before and during the drug infusion were SC5b-9 (Figure 5A), Bb (Figure 5B) and thromboxane (TX) B2 (Figure 5C). Starting from treatment 4, iC3b (Figure 5D) was added to the panel of complement activation tests. SC5b-9, Bb and iC3b are proteins from the C cascade. In vivo, complement activation triggers the production of TXA2 by platelets, which is in turn quickly converted to the more stable TXB2. Increases in TXB2 levels are therefore an indirect indication of the level of complement activation [37,38,39]. The results depicted in Figure 5 show variations in the different markers of complement activation between the beginning and the end of infusions. The results show that the values of 3 out of 4 markers (SC5b-9, iC3b and TXB2) remained within the normal physiological ranges defined as 0.1–0.49 µg/mL for SC5b-9 [40], below 1.2 µg/mL for Bb [40], 151.3 ± 15.5 µg/mL for iC3b [41], and 25–190 pg/mL for TXB2 [42] in plasma. The baseline levels of Bb fragment in the blood increased between the two series of treatments as seen by baseline levels of Bb before treatments 1–3 (0.73 ± 0.04 µg/mL) and before treatments 6–8 (1.43 ± 0.06 µg/mL). The values of Bb during the second set of treatments were above the normal physiological range (up to 1.5 to 2-fold increase, see Figure 5B) but this increase was not associated to any specific clinical symptoms and was therefore considered only subclinical manifestations of immune activation upon injection of NSSL-MPS.

To verify if the lack of complement activation we observed was due to IgG4 disease induced hypocomplementemia, the levels of C3 and C4 proteins were quantified in the serum of the patient before each session of treatment (i.e., before treatment 1 and 6). The values were found to be within the normal physiological range as defined by the hospital where the tests were performed. C3 concentration was 123.30 mg/dL and 95.49 mg/dL before the first treatment and the sixth treatment respectively, with a physiological range of 30–180 mg/dL, while C4 concentration was 14.99 mg/dL before the first treatment and 12.13 mg/dL before the 6^th^ treatment (normal range: 10–40 mg/dL). The pro-inflammatory cytokine IL-6 levels were quantified in the serum of the patient before the first treatment and during the 24 h that followed. All measured values of IL-6 were within the physiological normal range (defined as <10 pg/mL) but all the measured values from the patient’s serum were below the lowest concentration of the calibration curve (2 pg/mL for this test) and therefore are extrapolations from the lowest range of calibration. Before the beginning of infusion the IL-6 levels were estimated to be 1.07 pg/mL, 1 h after the beginning of NSSL-MPS infusion, the IL-6 level dropped to 0.41 pg/mL, and 7 h post-injection it reached 0.15 pg/mL. After 24 h no IL-6 could be detected.

### 2.4. Screening of Anti-PEG Antibodies in a Healthy Population

**Figure 6:**
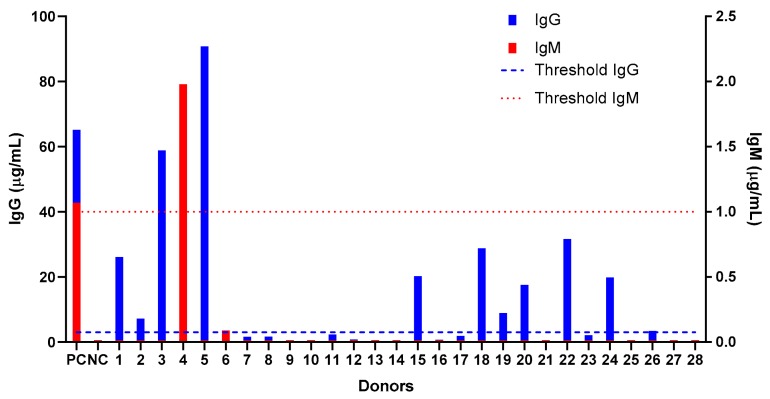
Screening of healthy donors for pre-existing antibodies against polyethylene glycol (anti-PEG IgG and IgM). Blood was collected from healthy donors (*n* = 28, 14 men and 14 women) and the sera were assessed for the presence of natural anti-PEG antibodies using a direct ELISA against immobilized PEG. The positive threshold was defined as 3 times the value of background for each antibody. Each positive result was confirmed by competition assay against PEGylated liposomes. Dashed line indicates the positive threshold for IgG and dotted line the positive threshold for IgM detection.

Prior to administering the NSSL-MPS to the patient, we tested the human anti-PEG detection assay in a small cohort of healthy donors (who were never treated with PEGylated drug) to assess their levels of pre-existing anti-PEG IgM and IgG antibodies. This is the continuation of a larger evaluation performed in Taiwan [12] and we used the same ELISA protocol as the one used in the Taiwanese study. We found that among 28 donors (14 men and 14 women), 11 (40%) had pre-existing IgG anti-PEG (5 women and 6 men), whereas only one donor (a woman) exhibited pre-existing anti-PEG IgM (see Figure 6). None of the donors had both IgG and IgM antibodies and, probably due to the small size of the population study, we found that the prevalence was different than the results published by Chen et al. [12] which shows a similar prevalence of anti-PEG IgM and IgG in the Taiwanese population (25% and 26.4% of pre-existing IgG and IgM respectively). 

### 2.5. Anti-PEG Antibodies in the Patient After Administration of NSSL-MPS

To assay the presence of pre-existing or elicited anti-PEG IgG and IgM in the blood of the patient before and after treatment, we performed the assay described previously [12] using chimeric antibodies directed against the PEG backbone. We found that before treatment, the patient didn’t have pre-existing anti-PEG antibodies. In addition, the patient’s serum was tested at different times after each treatment (24, 72 and 120 h post drug administration) and was found negative of any elicited anti-PEG IgG and IgM at all the tested times (see detailed results in Supplementary Materials Figure S4). 

### 2.6. Pharmacokinetics of Repeated Administrations of Liposomal MPS

**Figure 7:**
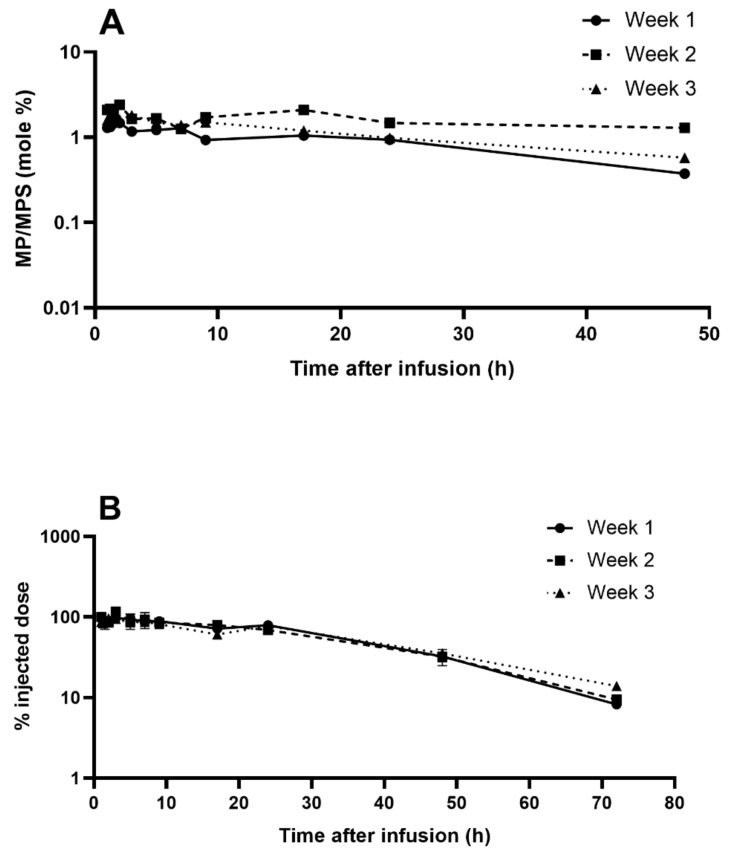
Levels of MPS and MP in the patient’s serum for treatments 1–3. (**A**) MP to MPS ratio (mole %) during time following 1-hour slow infusion of NSSL-MPS for treatment 1, 2 and 3. (**B**) Serum levels (as percent of injected dose) of methylprednisolone hemisuccinate following 1-hour slow infusion of NSSL-MPS for treatment 1, 2 and 3 (treatment with 50, 100 and 150 mg liposomal MPS respectively).

To complete the evaluation of IRs induced by NSSL-MPS, we performed a pharmacokinetics (PK) study to check if the patient demonstrated ABC phenomenon. We used liquid chromatography–mass spectrometry (LC-MS) to quantify the levels of both MPS and its active metabolite methylprednisolone (MP) in the patient’s serum upon repeated injections of escalating doses. The results for weeks 1, 2, and 3 (treatment with 50, 100 and 150 mg liposomal MPS respectively) are presented in Figure 7. The liposomal MPS demonstrated a prolonged circulation time with a terminal half-life (t_½_) ranging from 14–19 h, which is 56 to 76-fold longer than for intravenously administrated free MPS [43]. These values were calculated from the terminal slope of the logarithm of the total concentration–time curve. Similar t_½_ values of NSSL-MPS were previously reported in preclinical studies with three different mammalian species: mice (12 h), rats (11 h) and a dog(5.5 h) [23,24]. Area under the curve (AUC) values of 1262, 2163 and 3922 μg.h/mL (for treatment 1, 2 and 3 with 50, 100 and 150 mg liposomal MPS respectively) show dose dependence (AUC/Dose 22-26 h*µg/mL/mg) and linear kinetics. In the case of Doxil^®^, whose carrier has the same lipid composition as NSSL-MPS, drug clearance is predominantly determined by the liposomal carrier, and is very similar to the liposomal carrier’s PK [44,45]. PK studies of Doxil^®^ in rats showed that more than 93% of the doxorubicin measured in plasma remained encapsulated in liposomes [46]. In contrast, the release kinetics of MPS from the liposomes is faster with a significant contribution to drug clearance. In the case of NSSL-MPS, MPS release is characterized by a zero-order, slow drug release, as shown in vitro in human plasma and inflamed synovial fluid, and in vivo in rat’s systemic circulation and inflamed paw [23,24]. Free MPS is converted rapidly to the active pharmaceutical ingredient, methylprednisolone (MP) upon hydrolysis by the ubiquitous esterases found in biological fluids and inside cells [47], and therefore it can be assumed that most of the MPS observed in plasma is encapsulated in liposomes. MP is derived from MPS that was released from NSSL-MPS (free MPS) but it does not represent all that was released due to the fast clearance of both free MPS and MP [43].

The in vivo controlled release of MPS is apparent by monitoring the clearance of the NSSL, which have a half-life of ~34 h while the liposomal MPS half-life in rats was ~11 h [24]. This explains the significant difference with Doxil^®^, which due to lack of doxorubicin release during circulation in human blood [44,45] has a very long half-life of (50–80 h) compared to NSSL-MPS that has a half-life circulation of 14–19 h (Table 1). Still, circulation time of the liposomal MPS (14–19 h) is much longer than of the free MPS (0.25 h). The mean residence time (MRT) ranged between 25–27 h with a clearance rate of 40 ± 6 mL/h, which is 2250 fold slower compared to 90 ± 27 L/h for the free drug [43]. During the first 24 h after administration, a significant fraction of the drug (75 ± 5% of the injected dose) was retained in plasma. The serum concentration of total MP was on average 1.5% ± 0.2 (mole %) of the serum total concentration of MPS at each tested time and for all three treatments (Figure 7B), indicating that constant levels of the prodrug, MPS, turns into the actual active ingredient MP. These prolonged circulation times were evident and similar throughout 3 repeated administrations in the patient. Furthermore, treatments were administrated once a week. At this interval, the level of anti-PEG antibodies should already be detectable in case of elicited anti-PEG antibodies [15]. Table 1 presents PK parameters determined by the noncompartmental approach for each dose. The absence of ABC in this patient is supported by the lack of shortening of half-life and mean residence time (MRT) values, combined with good linear dose-response relationship between AUC and injected dose, as well as similar clearance and volume of distribution upon repeated administrations. This is in good agreement with the lack of elicited anti-PEG IgG and IgM antibodies. 

## 3. Discussion

The main objectives of this study were to evaluate the safety of our liposomal steroid (NSSL-MPS) that was fully characterized to determine physicochemical properties, toxicity and immunotoxicity in-vitro and to correlate in vitro data with its ability to elicit anti-PEG antibodies, increase complement-acivation bomarkers, induce ABC and cause Infusion Reactions in a “First in human” study. We also followed treatment efficacy. Altough this was not the main objective of this paper we can still say that our patient had progressed under several lines of treatment prior to initiating this compassionate use protocol of treatment with NSSL-MPS. We know that her disease stabilized and has been stable since treatment, with normalized levels of IgG4 and no growth of the mediastinal mass on MRI follow ups which we have obtained twice a year. We believe it is likely that the treatment with the steroidal nano-drug was efficacious in this outcome, but we are careful not to suggest that we have proof of that, since the patient received other treatments after this one.

Infusion reactions in general, and activation of the complement system in particular, are a growing hindrance to the development and use of a wide variety of nanodrugs. Thus, increasing efforts are required at early stages of development to detect and evaluate the potential of nanodrugs to be immuno-reactogenic. This requires identification of biomarkers that enable the prediction of infusion reactions and the use of in vitro tools in addition to the conventional physico-chemical, sterility and pyrogenicity characterization. Here we described and used such additional characterization assays as described in the Material and Methods above. In our study we tried to understand if in vitro tests performed *a priori* correlate with the in vivo changes in biomarkers levels during and following NSSL-MPS drug-product infusion, concomitantly with the presence of clinical signs of IRs. [10]. The initial observation reported in this study requires more clinical data to verify the correlation.

In vitro, at clinically relevant concentration, NSSL-MPS did not induce complement activation. At a very high concentration, NSSL-MPS demonstrated a small though measurable capacity to activate the complement system in vitro as seen by a slight increase in iC3b production when the NSSL-MPS were incubated in the plasma of healthy donors. However, even at the highest tested concentration, the complement activation by NSSL-MPS was lower than that caused by Doxil, which is successfully used in the clinic, despite being known to cause IRs in sensitive individuals. Doxil^®^ is included in the current analysis at the clinically relevant concentration to serve as an additional control [7]. NSSL-MPS did not induce a positive response in the remaining tests of our in vitro panel, demonstrating overall safe hematocompatibility profile. Specifically, it did not induce cytokines and chemokines release, did not activate leukocyte procoagulant activity, did not induce platelet aggregation, did not affect collagen-induced platelet aggregation and plasma coagulation time, and was not hemolytic. Even though these in vitro data generated using the blood of healthy individuals cannot be used *per se* to predict the incidence of IRs in patients, they provide information for the design of clinical studies. In the clinical study we detected insignificant complement activation which was not accompanied by the symptoms of IRs. Interestingly, in vivo iC3b levels were within the normal range and the only marker of complement activation tested that showed an increase above normal values was Bb, which suggests formation of convertase and the activation of the alternative pathway [48]. It was previously shown that an increase of Bb and other markers of complement activation up to 2 fold above the baseline level (and upper range of normal values) does not necessarily imply clinical infusion reactions, as was shown by Chanan-Khan and collaborators [49]. They measured the levels of SC5b-9 as an indication of complement activation in the plasma of patients treated with Doxil^®^, and showed that the correlation between complement activation and infusion reaction was the strongest when the levels of SC5b-9 were raised 4-fold or more above the upper threshold of normal plasma levels. A recent clinical study with a formulation of glutathione-PEGylated liposomes containing methylprednisolone [50] revealed an extremely high rate of infusion related reactions (89%) in patients following drug infusion. Analysis of complement activation factors in the blood of patients revealed drastic increase in C3a factor (spiking at>1500 ng/mL) and an increase in Bb levels of more than 16-fold in all patients receiving doses of 300 mg or more of liposomal MPS.

It has been shown that a non-negligible percentage of the patients with IgG4 RSD have hypocomplementemia marked by decreased CH50 [51] and/or low levels of the complement proteins C3 and C4 [52,53]. The prevalence of hypocomplementemia seems to be relatively high in patients with active disease and it has been reported that 20% of patients have C3 levels decreased to 59.4 mg/dL and C4 to 8 mg/dL [53]. We found that the patient had normal levels of both C3 and C4 proteins, and therefore the lack of immune response was not because of a deficiency in the complement system triggered by IgG4 RSD. Interleukin IL-6 is a primary mediator of both acute and chronic inflammation and is involved in the pathogenesis of many chronic inflammatory diseases [54,55]. It has been found that in some cases of IgG4 RSD, patients have elevated IL-6 levels [56,57] but that was not the case for the patient in our study. Moreover, the administration of NSSL-MPS decreased IL-6 to non-detectable levels even after 24 h, as seen previously in in vivo studies [27].

The lack of production of anti-PEG antibodies following NSSL-MPS administration was very encouraging because of their contribution to complement activation and modification of the PK through the ABC. The absence of elicited anti-PEG antibodies was further supported by the lack of ABC and the long circulating time of NSSL-MPS in plasma observed in the PK study for all the administrations (here we show the data for weeks 1–3, escalating doses from 50 to 150 mg). The PK results show that a significant level of the drug was retained in plasma for at least 24 h after the 2nd and 3rd administrations and there were still detectable levels of the drug after 3 days (~10% of the injected dose), demonstrating prolonged circulation half-life (56–76 fold higher compared to non-liposomal drug) and dose-dependent serum levels. In addition, despite repeated administration and prolonged circulating time of the glucocorticoids, the liposomal formulation provided good protection from the side-effects inherent to glucocorticoids treatment. Glucose levels remained stable throughout the treatments and the increase in WBC numbers was only transient. A rapid and sharp increase (up to approximately 22 k/µL) in leukocytes is a well-known secondary effect of treatment with glucocorticoids [58,59], although it usually lasts longer than what was observed in the patient, and the WBC count doesn’t return to baseline [59]. Likewise, the administration of glucocorticoids triggers a transient decrease in lymphocytes and an increase in neutrophils and both effects were observed in the patient, but the values returned to baseline within a week. These changes in cell counts could be caused by the presence of small amounts of free MPS contained in the NSSL-MPS formulation (~0.5 mg/mL, so approximately 6, 12 and 18 mg for the treatment with 50, 100 and 150 mg MPS respectively) but they are more probably caused by the administration of corticosteroids (50 mg) immediately prior to the infusion of NSSL-MPS as part of the pre-treatment to avoid infusion reactions (see the methods section). It is hard to evaluate to what extent the pre-medication administered to the patient played a role in avoiding IRs, but from an ethical point of view pre-treatment was mandatory to reduce the potentially life-threatening side effects of IRs. In addition, with other nano-drugs, it is known that despite pre-medication some patients still experience IRs to several types of drugs such as antibodies [60] but also anticancer therapy [34], or lately a new formulation of glutathione-PEGylated liposomal methylprednisolone [50]. Therefore, pre-medication was, and should still be administered, as a mean of prevention even though our formulation didn’t give any signs of complement activation in the patient.

In conclusion, we presented herein a set of tests that are simple yet comprehensive for the early detection of patients at high risk of developing IRs upon injection of PEGylated nanodrugs. These tests were performed before and during treatment with a PEGylated liposomal steroid formulation and included immunoreactivity tests of the drug-product formulation in vitro, but especially in vivo tests performed on the patient’s plasma for markers of complement activation and the presence of anti-PEG IgM and IgG. We completed our study with a PK study to confirm the absence of ABC (which we did not expect since we didn’t find anti-PEG antibodies) and other secondary effects related to the drug encapsulated (glucocorticoid). In our study, the patient didn’t develop IRs during the 8 injections of the drug and we cannot say, due to the unique character of the treatment that was a compassionate use of the drug, if the reason is because of the low immunogenicity of the formulation, low risk of the patient to IRs and/or the effect of the pre-treatment (or a combination of all these parameters). Therefore, we couldn’t evaluate the correlation between the biomarkers’ quantification and the clinical signs (and their severity) of IRs. But we were able to detect small variations that were associated to sub-clinical immune manifestations. Here, we demonstrate the ability to perform this battery of in vitro and in vivo assays in humans. It is possible that in addition to perform the in vitro test in healthy donors, it will be informative to performing it also in the plasma of patients expected to get treatment with the nano-drug. When these assays will be applied to a larger cohorts of patients they may enable in vitro–in vivo correlation as well as determining the threshold levels of the outcome of the in vitro and in vivo biomarkers’ assays from which a prediction of the potential of nano-drug to induce severe IRs can be evaluated.

## 4. Material and Methods

### 4.1. Preparation of the NSSL-MPS

NSSL-MPS were prepared as previously described [25]. Briefly, freeze-dried hydrogenated soybean phosphatidylcholine (HSPC), cholesterol and 1,2-distearoyl-sn-glycero-3-phosphoethanolamine-*N*-(methoxy(polyethylene glycol)-2000 Da) sodium salt (DSPE-PEG2K) mixed at 3:1:1 weight ratio (or 56.6:38.1:5.3 mole ratio; Lipoid GmbH, Ludwigshafen, Germany) were dissolved in absolute ethanol and this solution was mixed with 250 mM calcium acetate in order to hydrate the lipids and form a dispersion of multilamellar liposomes (MLV). These MLV were then downsized by sequential extrusion steps. Transmembrane gradient of calcium acetate was created by diafiltration and the nano-liposomes were then remotely loaded with MPS sodium succinate (Solu-Medrol, Pfizer: NYC, NY, USA). See more details in Avnir et al., [23,24].

### 4.2. Physicochemical Characterization of NSSL-MPS

Liposomes size and size distribution were measured by dynamic light scattering (NanoZetasizer, Malvern, Worcestershire, UK). The Z-average of the preparation was found to be 79 nm with a polydispersity index (PDI) of 0.03. The zeta potential was -17mv measured in 1.5mM sodium nitrate in iso-osmotic 10% sucrose (pH 5.5) while in 150 mM sodium nitrate (which represent plasma ionic strength) it has a value of ~1.0 mV (namely being chargeless). The phospholipid concentration was 43 mg/mL as determined using the modified Bartlett procedure [61] and 42.7 mg/mL as determimed by HPLC equiped with ELSD detector (Buchi, Alltech Associatesm Flawil, Switzerland) [62]. The concentrations of total and intraliposomal MPS, (obtained after treatment with Dowex 2X-800 anion exchanger at pH 5, which binds all free drug) and MPS hydrolysis products were quantified using the previously developed HPLC protocol described in detail by Avnir et al. [24]. Total MPS concentration was 5.2 mg/mL of which 88% of the drug was encapsulated inside the liposomes. Lipid to drug ratio was 8.21. The pH of the final formulation was 6.57 and the osmolality 391 mOsm/kg. This drug-product was sterile and apyrogenic. Pyrogenicity was determined by both Limulus Amebocyte Lysate test (LAL) test performed by HyLabs, Israel (λ < 1.2 EU/mL) and by rabbits test. The rabbits test was performed by Envigo, Israel according to USP 2015, 38-NF32. Sterility was tested by membrane filtration (AMINOLAB, Nes Ziona, Israel).

### 4.3. In Vitro Complement Activation by the NSSL-MPS

The evaluation of in vitro activation of the complement was performed as described previously [63]. To monitor complement activation, C3 split product iC3b was chosen because the three main known pathways of the complement activation converge on this protein and iC3b is one of the stable split products generated during C3 cleavage. Briefly, the in vitro activation of the complement cascade was measured by quantification of the complement split product iC3b generated in pooled human plasma (collected in blood collection tubes containing sodium citrate) exposed for 30 min at 37 °C to diluted NSSL-MPS using a commercial kit (MicroVue iC3b EIA, Quidel, A006). The increase in iC3b was compared to that caused by Cobra Venom Factor (CVF) and Cremophor-EL, and sterile Ca^2+^/Mg^2+^ free PBS was used as the negative control.

### 4.4. In vitro Cytokines Release

The cytokines/chemokines release from whole blood cultures were measured as described previously [64]. Briefly, different dilutions of NSSL-MPS (0.304, 1.52, 7.62, 76.2 µg/mL MPS), positive and negative controls were incubated for 24 h (37 °C, 5% CO2 incubator, NuAire, Plymouth, MN, USA) in whole blood of at least 3 different healthy donors diluted 1:4 in complete culture media (RPMI with 10% heat inactivated FBS, 2 mM L-glutamine, 100 U/mL penicillin, and 100 μg/mL streptomycin). After the incubation, the cultured blood samples were collected and centrifuged at 18,000× *g* for 5 min. The supernatants were analyzed by multiplex ELISA (Quansys Biosciences, Logan, UT, USA) to determine levels of individual cytokines.

### 4.5. Platelet Aggregation and Collagen-Induced Platelet Aggregation

The capacity of NSSL-MPS to induce platelet aggregation and modify collagen-induced platelet aggregation were assessed according to the protocol described earlier [65]. Briefly, NSSL-MPS at different concentrations (0.304, 1.52, 7.62 or 76.2 µg/mL MPS) were incubated in platelet-rich plasma for 15 min at 37 °C. Platelet aggregation was monitored in real time using light transmission aggregometry (Chronolog, Havertown, PA, USA).

### 4.6. Leucocyte Pro-Coagulant Activity

Peripheral blood mononuclear cells (PBMC) isolated from healthy blood donor volunteers and HL-60 cells were incubated (24 hs for PBMC and 5 hrs for HL-60 cells) with NSSL-MPS at 0.304, 1.52, 7.62 or 76.2 µg/mL MPS. Isolated cells from the different conditions were then used to initiate plasma coagulation using the protocol described earlier [65]. Briefly, after the incubation with the particle, the cells were washed and added to the autologous plasma. The coagulation time was monitored in prothrombin assay using STArt4 hemostasis analyzer (Diagnostica Stago, Parsippany, NJ, USA)

### 4.7. In Vitro Evaluation of NSSL-MPS Hemolytic Activity

As part of the toxicity characterization NSSL-MPS was evaluated for its hemolytic activity as described previously [66]. Briefly, NSSL-MPS and controls were incubated in whole blood at 37 °C for 3 h (± 15 min). Undamaged erythrocytes were precipitated by centrifugation (15 min at 800 × *g*) and the collected plasma was incubated with Drabkin’s reagent so that the free hemoglobin in the plasma, released by the damaged erythrocytes, was converted to cyanmethemoglobin, a stable derivative of hemoglobin. The amount of cyanmethemoglobin in the supernatant was quantified by spectrophotometry at 540 nm. This measured absorbance was compared to a standard curve to determine the concentration of hemoglobin in the supernatant. The measured hemoglobin concentration was then compared to the total hemoglobin concentration to obtain the percentage of nanoparticle-induced hemolysis [67].

### 4.8. Patient’s Treatment with NSSL-MPS

The patient’s treatment consisted of two courses of injections set 6 months apart: the first one was five-week long and the second three-week long. In the first part of the treatment, escalated doses of 50, 100, 150 mg MPS in NSSL-MPS were administered I.V. once a week for 5 consecutive weeks (the highest dose of 150 mg was administered for the last 3 weeks). In the second course, two doses of 225 mg, then one dose of 300 mg MPS in NSSL-MPS were administered I.V. once a week for three consecutive weeks. Before each NSSL-MPS administration, the patient received appropriate pre-medication consisting of a cocktail of corticosteroids, acetaminophen and H2 blockers in order to decrease the risk of infusion reactions (protocol similar to the pre-medication protocol required prior to Onpattro^®^ administration [68]). The different doses of drug were diluted in 5% Dextrose for injection for a total fixed volume of 250 mL/treatment. Based on Doxil experience, diluted NSSL-MPS was administered at a slow rate of 0.4 mg/min for approximately 15 min. If no clinical sign of infusion-related reaction was observed, the rate of infusion was then increased to complete the administration over a period of 1 hour ranging from 1 mg/min (treatment with 50mg liposomal MPS) to a maximum of 6.3 mg/min (treatment with 290 mg liposomal MPS). Clinical parameters were monitored during administration and for at least 2 h post administration (body temperature, pulse, blood pressure, oxygen saturation and respiratory rate) and any other clinical sign mentioned by the patient was recorded.

### 4.9. Blood Sampling

Plasma samples were collected immediately prior to (T_0_) and during the drug infusion (10, 20, 30 and 60 min after the beginning of drug infusion) to be tested for in vivo complement activation. The blood samples for plasma analysis were collected in K_2_EDTA collection tubes (lavender caps, Vacutainer, BD) and immediately put on ice. They were centrifuged as soon as possible after collection (960 × *g* for 10 min at 4 °C) and plasma was collected and aliquoted under sterile conditions and immediately frozen in dry ice.

Serum samples were used for the detection of anti-PEG antibodies (before the study and approximately 24, 72 and 120 h after each drug administration) and for the PK study (1, 2, 3, 5, 7, 9, 17, 24 and 48 h after the beginning of the infusion). Serum samples were withdrawn for treatment 4 only at baseline per patient’s request (no PK study was performed for this treatment). All blood samples for serum analysis were collected in serum collection tubes (yellow caps Vacutainer, BD) and kept at room temperature (RT) for 30–60 min then centrifuged (960 × *g* for 10 min, RT). The sera collected were aliquoted, frozen and kept at −80 °C until analysis. An additional blood sample was collected before each treatment for glucose measurement and complete blood count.

### 4.10. Anti-PEG Detection Assays in Human Plasma

Chimeric anti-PEG antibodies (c3.3, an IgG anti-PEG, and cAGP4, an IgM anti-PEG) were obtained from our collaborators (Roffler et al.) who developed them [69,70]. The patient’s serum was assayed for the presence of anti-PEG IgG and IgM in a direct ELISA against immobilized PEG as described previously in details [12]. Briefly, the patient’s serum samples were diluted 25-fold in 2% (*w/v*) powdered skim milk in PBS (Dulbecco’s phosphate-buffered saline, Thermo Fisher Scientific). Two additional 2-fold serial dilutions were made in dilution buffer (4% human negative serum in 2% skim milk in PBS). Standard curves were obtained by serial dilutions (3-fold) of chimeric anti-PEG antibodies (c3.3 or cAGP4) starting at 2.5 or 2 μg/mL, respectively in dilution buffer. The patient’s serum samples at dilutions of 25, 50, and 100-fold and the antibody standards were incubated in duplicate for 1 h RT in Maxisorp 96-well microplates previously coated with 0.5 μg/well NH2-PEG10K-NH2 then blocked with skim milk 5% in PBS. Unbound antibodies were washed with 0.1% CHAPS (3-[(3-cholamidopropyl)-dimethylammonio]-1-propanesulfonate) in PBS, then PBS only. Horseradish peroxidase (HRP)-conjugated secondary antibodies (goat F (ab′)2 anti-human IgG Fc or goat F (ab′)2 anti-human IgM) were added to the IgG or IgM detection plates, respectively, for 1 h RT. The plates were washed and incubated with ABTS substrate for 30 min RT. The absorbance (405 nm) in the wells was measured using a BioTek Synergy™ 4 Hybrid Microplate Reader (Winooski, VT, USA). Positive responses were defined as samples with absorbance values at least 3 times greater than the mean background absorbance (dilution buffer). The relative concentrations of anti-PEG IgG or IgM in positive samples were calculated by comparison with c3.3-IgG or cAGP4-IgM standard curves, respectively. Positive samples were confirmed by a PEG competition assay as described in [12]. 

### 4.11. In Vivo Quantification of Complement Activation Markers During NSSL-MPS Infusion

The levels of different plasmatic markers of complement activation were quantified in the plasma of the patient collected during the drug infusion: iC3b, Bb, SC5b-9 (The Terminal Complement Complex) as well as thromboxane B2 (TXB2) levels. The measurements were performed using commercial Enzyme Immunoassays kits (Quidel, San Diego, CA, USA): MicroVue iC3b EIA (Quidel, A006), MicroVue Bb Plus EIA (Quidel, A027), MicroVue SC5b-9 Plus EIA (Quidel, A020) and 11-dehydro Thromboxane B2 ELISA Kit (Cayman Chemical, Ann Arbor, MI, USA, #19510). C3 and C4 were quantified by turbidimetry in the serum of the patient before each cycle of weekly treatments (i.e. before treatment 1 and 6) at Hadassa Ein Karem Hospital (Jerusalem, Israel), using a Cobas 6000 Analyzer (Roche Diagnostics, Basel, Switzerland). IL-6 was quantified using a chemiluminescent enzyme immunometric assay (Immulite 1000, Siemens) by Hadassa Ein Karem Hospital (Jerusalem, Israel).

### 4.12. Pharmacokinetic Study

To evaluate the levels of MPS and MP after infusion at each blood collection time, sera were prepared and analyzed in duplicate. To 100 µL of serum, 30 ng (10 μL of a methanolic solution at 30 µg/mL) of the internal standard hydrocortisone succinate (HC) and budesonide (BUD) were added before each analysis. Afterwards, samples were extracted with 6 mL of ethyl acetate, evaporate to dryness with filtrated air at 30 °C using Eppendorf Concentrator 5301 (Eppendorf AG, Hamburg, Germany) and re-dissolved in 100 μL methanol. The chromatography was performed under reverse phase conditions using a Shimadzu (Kyoto, Japan) UHPLC System as described in more details in the Supplementary materials. Results are presented as the mean ± STDEV. The large number of data points allowed for a detailed data analysis and NCAanalysis using Phoenix ® WinNonlin ® www.certara.com (Certa USA, Inc., Princeton, NJ, USA).

## Figures and Tables

**Table 1:** Pharmacokinetics parameters.

Treat-ment #	Dose Liposomal MPS	R^2^	Terminal Half-Life	C_max_	C_max_/Dose	AUC_last_	AUC_last_/Dose	Cl_obs_	MRT_last_
	mg		h	µg/mL	µg/mL/mg	h*µg/mL	h*µg/mL/mg	mL/h	h
**1**	50	0.98	14	35	0.7	1262	25	38	25
2	100	0.98	17	68	0.7	2163	22	46	27
3	150	0.998	19	108	0.7	3922	26	35	27

* The calculated plasma volume for a female patient weighing 55 kg, measuring 164 cm and with 40% hematocrit is ~2 liters. AUC, Area Under the Curve; Cl, Clearance; MRT, Mean Residence Time; Vss, Volume of Distribution.

## Supplementary Materials

The following are available online. Figure S1: Plasma coagulation times in presence of NSSL-MPS. Figure S2: Variations of blood glucose levels during and after treatment with increasing doses of NSSL-MPS, ranging from 50 to 300 mg MPS. Figure S3: Changes in blood cell counts after treatment with different doses of NSSL-MPS ranging from 50 (treatment 1) to 300 mg MPS (treatment 8). Figure S4: Anti-PEG antibodies detection in the serum of the patient before and after treatments.

## Abbreviations

AUC: Area under the curve; ABC, Accelerated Blood Clearance; C, Complement; HSR, hypersensitivity reactions; IgG4 RSD, IgG4-related sclerosing disease; IR, infusion reactions; LC-MS, Liquid chromatography–mass spectrometry; MCP, Monocyte chemoattractant protein; MIP, Macrophage inflammatory protein; MP, methylprednisolone; MPS, methylprednisolone hemisuccinate; NCL, Nanotechnology Characterization Laboratory; NSSL-MPS, nano-sterically stabilized liposomes loaded with MPS; PEG, polyethylene glycol; PK, pharmacokinetics; RANTES, Regulated on Activation, Normal T Cell Expressed and Secreted; TX, thromboxane; WBC, white blood cells.
