# Supplementary material for: PEGylated Liposomal Methyl Prednisolone Succinate does not Induce Infusion Reactions in Patients: A Correlation Between in Vitro Immunological and in Vivo Clinical Studies
Source: Molecules. 2020 Jan 28;25(3):558. doi: 10.3390/molecules25030558 (PMC7037198; doi:10.3390/molecules25030558)
Supplement: Supplementary file 1 [file molecules-25-00558-s001.pdf]

molecules

MDPI

Research Article

SUPPLEMENTARY INFORMATION

PEGylated liposomal methyl prednisolone succinate does not induce infusion reactions in patients: a correlation between in vitro immunological and in vivo clinical studies

Yaelle Bavli[a], Bing-Mae Chen[b], Steve R. Roffler[b], Marina A. Dobrovolskaia[c], Eldad Elnekave[d*], Shifra Ash[e], Yechezkel Barenholz[a†*] and Keren Turjeman[a†]

[a] Laboratory of Membrane and Liposome Research, Department of Biochemistry, Institute for Medical Research Israel-Canada, Hebrew University-Hadassah Medical School, Jerusalem, Israel.

[b] Institute of Biomedical Sciences, Academia Sinica, Taipei 11529, Taiwan.

[c] Nanotechnology Characterization Laboratory, Cancer Research Technology Program, Frederick National Laboratory for Cancer Research sponsored by the National Cancer Institute, Frederick, MD, USA.

[d] Davidoff Cancer Institute, Rabin Medical Center, Petach Tikva, Israel.

[e] Division of Oncology, Schneider Children's Hospital, Petach Tikva, Israel.

† Equal senior authors

* Corresponding authors (eldad.elnekave@gmail.com, chezyb1@gmail.com)

Instruments used for the quantification of MPS and MP in the serum of the patient

UHPLC instrument: The chromatography was performed under reverse phase conditions using a Shimadzu (Kyoto, Japan) UHPLC System, series Nexera, consisting of a Shimadzu CBM-20A LITE controller, two Shimadzu LC-30AD pumps, including a Shimadzu Prominence DGU-20A5R degasser, a Shimadzu SIL-30AC autosampler and a Shimadzu CTO-20AC column oven. The chromatographic separations were performed on a Kinetex™ (Phenomenex, Torrance, CA, USA) column (Kinetex C18, 2.6 µm particle size, 100 Å pore size, 100 x 2.1 mm), protected by a SecurityGuard™ (Phenomenex, Torrance, CA, USA) ULTRA cartridge (C18, 4 x 2 mm). The injection volume was 5 µL, the oven temperature was maintained at 40°C. The chromatographic separation was achieved using a linear gradient program at a constant flow rate of 0.45 mL/min over a total run time of 11 min. The first 1.0 min of the column effluent were diverted to waste. Methanol was used for washing the needle prior to each injection cycle. All samples were analyzed in duplicate.

MS/MS conditions

MPS and hydrocortisone hemisuccinate (HS) internal standard (IS) and MP and budesonide (BU) IS were detected by a Sciex (Framingham, MA, USA) Triple Quad™ 5500 mass spectrometer in positive ion mode using electrospray ionization (ESI) and multiple reaction monitoring (MRM) mode of acquisition. Air was produced (SF 2FF compressor, Atlas Copco, Belgium) and purified using an NM20Z nitrogen generator (Peak Scientific, Inchinnan, Scotland). Purified air was used as source and exhaust gases and purified nitrogen as curtain and collision gases. A receiver was placed between the compressor and the nitrogen generator for a large and stable supply of air. Optimal detection conditions were determined by constant infusion of 100 ng/mL solutions of the compounds in 1:1 Acetonitrile:water using the integrated syringe pump (5 µL/min). Transitions were selected and their settings were determined using Analyst Software in compound optimization mode.

SUPPLEMENTARY FIGURES

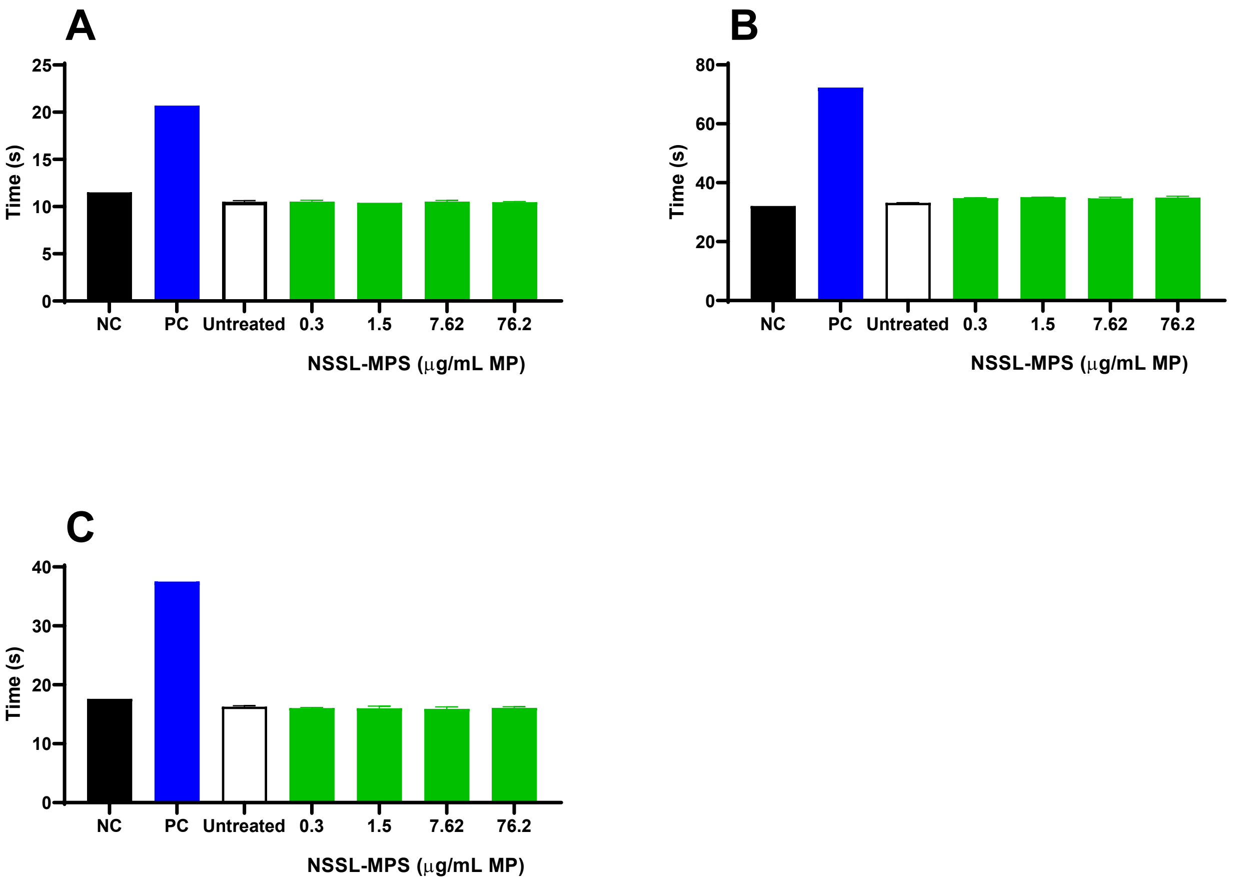

Figure S1. Plasma Coagulation Times in presence of NSSL-MPS. Prothrombin Time (A), Activated Partial Thromboplastin Time (B) and Thrombin Time (C) were measured incubation with different concentrations of NSSL-MPS. N, normal plasma standard; P, abnormal plasma standard.

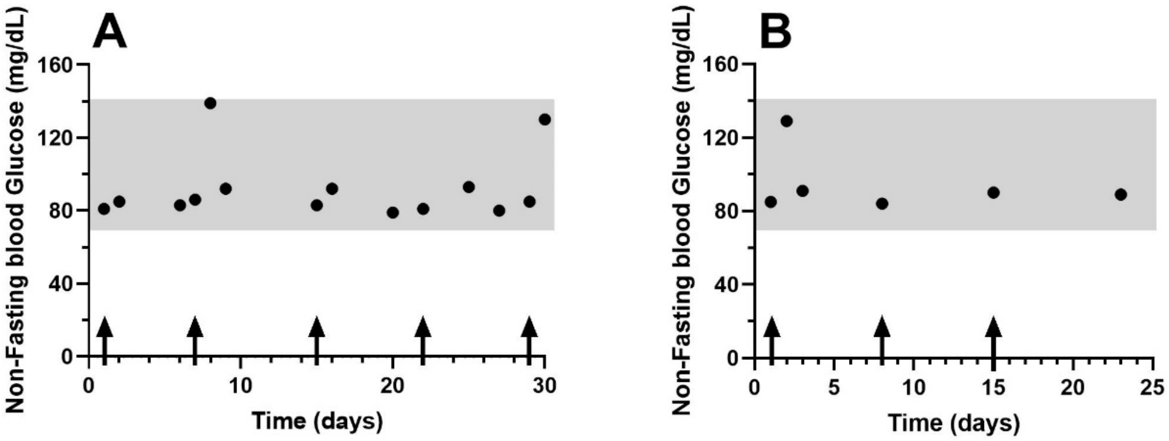

Figure S2. Variations of blood glucose levels during and after treatment with increasing doses of NSSL-MPS, ranging from 50 to 300 mg MPS. (A) Blood glucose levels during treatments 1 to 5 (respectively 50, 10, 150, 150 and 150 mg MPS). The measurements of days 8 and 30 were performed between 1-2 hours post-prandial. (B) Blood glucose levels during treatments 6 to 8 (225, 225 and 300 mg MPS). Each vertical arrow indicates the day of treatment and the grey shaded area the normal range of values for post-prandial blood glucose values.

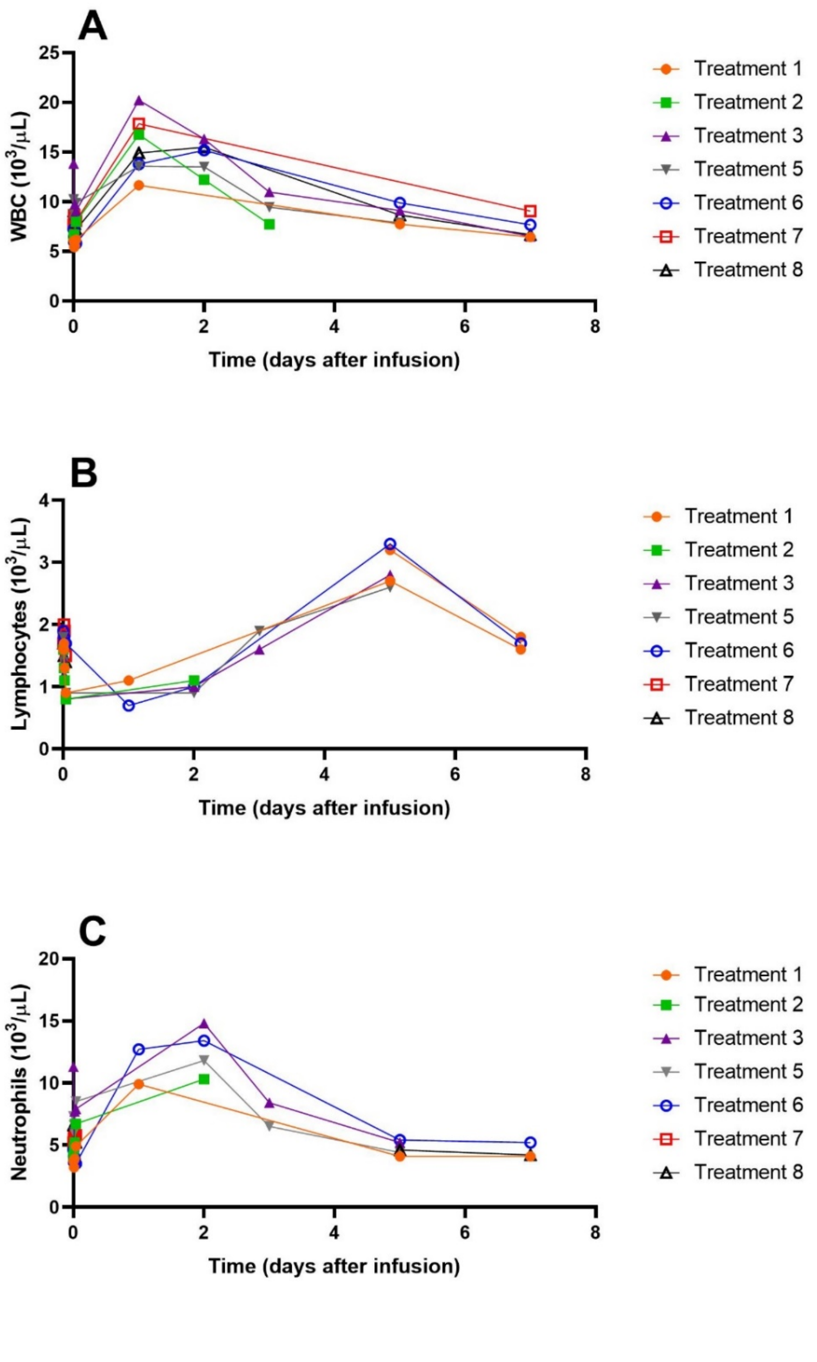

Figure S3. Changes in blood cell counts after treatment with different doses of NSSL-MPS ranging from 50 (treatment 1) to 300 mg MPS (treatment 8). Changes in Leukocytes (A), Lymphocytes (B) and Neutrophils (C). WBC, White Blood Cells.

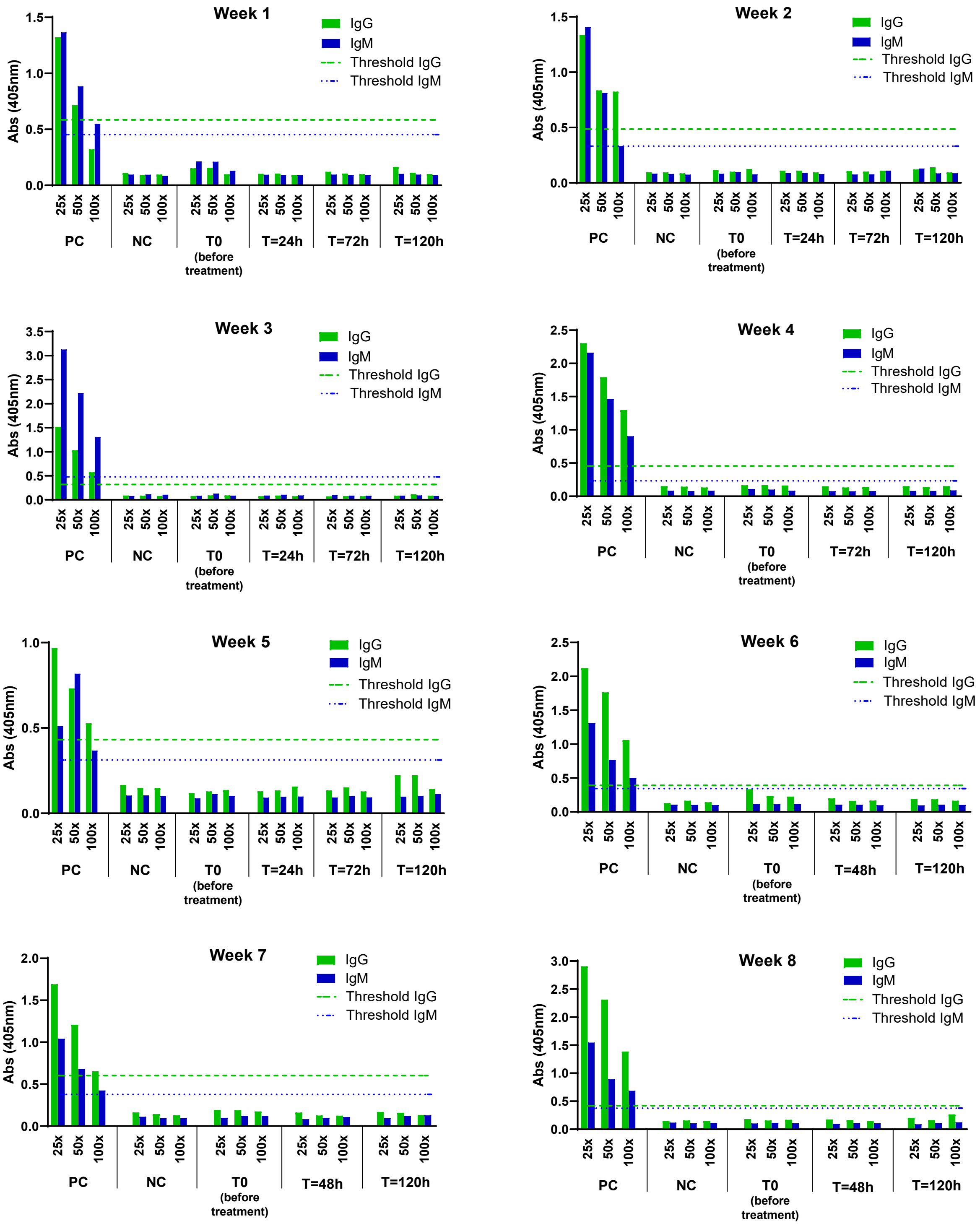

Figure S4. Anti-PEG antibodies detection in the serum of the patient before and after treatments. Blood samples were collected at different time-points. The sera were extracted and assayed for the presence of anti-PEG IgG and IgM with direct ELISA against immobilized PEG. The positive threshold was defined as 3 times the value of background for each antibody. Each positive result was confirmed by a competition assay.
